# Predictors of Long COVID in Adults With Untreated Mild-to-Moderate Coronavirus Disease 2019

**DOI:** 10.1093/ofid/ofag577

**Published:** 2026-11-13

**Authors:** Teresa H Evering, Pooja T Saha, Carlee Moser, Nikolaus Jilg, Rachel A Bender Ignacio, Prasanna Jagannathan, Katya Corado, Kevin Wongsodirdjo, Justin Ritz, Jonathan Z Li, Davey M Smith, Judith S Currier, Michael D Hughes, Kara W Chew

**Affiliations:** Department of Medicine, Weill Cornell Medicine, New York, New York, USA; Center for Biostatistics in AIDS Research, Harvard T.H. Chan School of Public Health, Boston, Massachusetts, USA; Center for Biostatistics in AIDS Research, Harvard T.H. Chan School of Public Health, Boston, Massachusetts, USA; Department of Medicine, Massachusetts General Hospital and Brigham and Women's Hospital, Harvard Medical School, Boston, Massachusetts, USA; Department of Medicine, University of Washington, Seattle, Washington, USA; Vaccine and Infectious Disease Division, Fred Hutchinson Cancer Center, Seattle, Washington, USA; Department of Medicine, Stanford University School of Medicine, Stanford, California, USA; Department of Medicine, Lundquist Institute at Harbor-UCLA Medical Center, Torrance, California, USA; Center for Biostatistics in AIDS Research, Harvard T.H. Chan School of Public Health, Boston, Massachusetts, USA; Center for Biostatistics in AIDS Research, Harvard T.H. Chan School of Public Health, Boston, Massachusetts, USA; Department of Medicine, Brigham and Women's Hospital, Harvard Medical School, Boston, Massachusetts, USA; Department of Medicine, University of California San Diego, La Jolla, California, USA; Department of Medicine, David Geffen School of Medicine at UCLA, Los Angeles, California, USA; Center for Biostatistics in AIDS Research, Harvard T.H. Chan School of Public Health, Boston, Massachusetts, USA; Department of Medicine, David Geffen School of Medicine at UCLA, Los Angeles, California, USA

**Keywords:** clinical trial, long COVID, postacute sequelae of SARS-CoV-2 infection (PASC), post-COVID condition, sex-stratification

## Abstract

**Background:**

We evaluated risk factors for long COVID (LC) in the ACTIV-2/A5401 trial, with a focus on understanding the impact of sex.

**Methods:**

Findings were analyzed in nonhospitalized adults with coronavirus disease 2019 (COVID-19) receiving placebo within 10 days of symptoms. LC was defined as self-reporting COVID-19–related symptoms at study week 24 in a diary. Demographics and clinical variables, nasal viral RNA (vRNA), and inflammatory/coagulation markers were evaluated as predictors of LC, using modified Poisson regression models.

**Results:**

Among 545 participants enrolled in January–August 2021 with a week 24 diary, the median age at entry was 44 years, 53% were female, 13% Black, 53% Hispanic/Latino, 83% US enrolled, 15% vaccinated, and 52% severe acute respiratory syndrome coronavirus 2 (SARS-CoV-2) seropositive. Seventy-four (14%) had LC (19% of female and 7% of male participants). Female sex was strongly associated with LC (adjusted risk ratio, 2.64 [95% confidence interval, 1.58–4.42]). Other predictors included US enrollment, higher total symptom score at entry, presence of symptoms at the end of the acute period (days 22–28), 28-day area under the curve of vRNA levels, and higher day 28 C-reactive protein and fibrinogen. The effect of sex persisted with adjustment for the other variables, and associations with symptoms and fibrinogen level varied by sex.

**Conclusions:**

In untreated adults with COVID-19, female sex, US enrollment, symptom and vRNA burden, and inflammatory/coagulation measures were associated with increased LC risk, with some associations varying by sex. The association with female sex was not significantly attenuated by adjustment for the assessed biomarkers. These data support sex-stratified investigations of LC, as well as a potential role of thromboinflammation.

**Clinical Trials Registration:**

ClinicalTrials.gov NCT04518410.

The long-term health impacts of coronavirus disease 2019 (COVID-19) continue to expand [1]. Many with severe acute respiratory syndrome coronavirus 2 (SARS-CoV-2) infection will experience persistent symptoms after acute infection, termed “long COVID” (LC) [2, 3]. Exact definitions of LC vary [2, 4–6]; however, this heterogeneous disorder can affect multiple organ systems with a variety of respiratory, cardiovascular, hematologic, immunologic, and neuropsychiatric symptoms [7–9].

Several characteristics have been associated with increased prevalence of LC including advanced age [10, 11], obesity [12, 13], poor pre–COVID-19 health [11], severe acute COVID-19 illness, prolonged hospitalization, and requirement for intensive care [14, 15]. Several studies have also suggested sex-based differences in LC risk [11, 16–20], but mechanisms underlying these differences remain poorly understood. Most prior studies identifying LC risk factors have relied on retrospective or observational designs, and few prospective trials have included prespecified LC end points alongside concurrent collection of virologic and inflammatory biomarkers during the acute infection period. There is an ongoing need to identify risk factors for and understand mechanisms of LC in order to improve its prevention and treatment [21].

In the current analysis, using data from nonhospitalized adults with acute COVID-19 randomized to placebo in the ACTIV-2/A5401 trial [22], we assessed associations between LC symptoms about 6 months after infection and demographic and clinical characteristics, as well as virologic, serologic, inflammatory and coagulation biomarkers. We focused on factors that might explain the higher risk of LC among female participants. Data were collected within a rigorous prospective blinded platform trial designed to address prespecified LC end points.

## METHODS

### Study Design and Participants

ACTIV-2/A5401 evaluated the safety and efficacy of investigational agents for the treatment of nonhospitalized adults with mild-to-moderate COVID-19 in a phase 2/3 adaptive platform trial (NCT04518410). Eligible participants were nonhospitalized adults (aged ≥18 years) within 10 days of symptom onset with a positive SARS-CoV-2 antigen or nucleic acid test result within 7 days before study entry and ongoing COVID-19 symptoms within 48 hours before entry. Partway through the trial, a long-term study diary was introduced to collect participant self-reported symptoms and ascertain the presence of LC. Participants eligible for this analysis were those randomized to placebo across the platform between January and August 2021. The analysis was restricted to placebo recipients to evaluate predictors of LC in the absence of potential treatment effects from investigational agents. Participants enrolled at 1 of 7 sites identified by the study sponsor as having data integrity concerns were excluded from analysis. The analysis was further restricted to participants with responses to the long-term symptom diary at week 24, the time point used to define LC in our analysis.

### Assessments: Participant-Completed Symptom Diaries

Participants completed a 13-symptom diary daily from enrollment (day 0) through day 28, as previously described for acute symptoms [23]. Participants graded each symptom as “absent,” “mild,” “moderate,” or “severe” and were instructed to report the worst severity over the preceding 24 hours by self-assessment. A total symptom score (with values between 0 and 39) on day 0 was calculated as the sum of the individual scores for the 13 symptoms, with absent scored as 0, mild as 1, moderate as 2, and severe as 3. A long-term symptom diary, also described elsewhere [18, 24], was completed 24 weeks after study entry, assessing the same 13 acute symptoms plus 14 additional LC symptoms selected based on the available literature at the time (27 total individual symptoms). Participants also reported the overall severity of their symptoms over the past 4 weeks by self-assessment [18, 24]. For both diaries, participants were not asked to assess relatedness of individual symptoms to COVID-19.

### Virology

Participant-collected anterior nasal swabs were obtained using standardized swabs and collection procedures daily on days 0–14 and day 28 in phase 2 and on days 0, 3, 7, 14, and 28 in phase 3 for quantitative SARS-CoV-2 RNA PCR (collection and analysis methods described elsewhere) [23]. As a measure of viral burden over the 28-day acute period of follow-up, the area under the curve (AUC) of log_10_ SARS-CoV-2 RNA values over time above the assay lower limit of quantification (LLoQ; 2 log_10_ copies/mL) was calculated.

### Serology, Inflammation and Coagulation Biomarkers

Antinucleocapsid (anti-N) and antispike (anti-S) binding antibodies in serum were measured on day 0 by a central laboratory; serostatus was defined as positive if either or both anti-N and anti-S were positive and as negative if both were negative. Serum inflammatory and coagulation markers including C-reactive protein (CRP), ferritin, D-dimer, lactate dehydrogenase (LDH), prothrombin time (PT)/international normalized ratio (INR), activated partial thromboplastin time (aPTT), and fibrinogen were measured in real time by a central clinical laboratory at days 0 and 28, per the manufacturers' protocols (see Supplementary Methods).

### Outcome Measures

LC, the primary outcome for this analysis, was defined as the overall presence of COVID-19 symptoms (any mild, moderate, or severe) in the 4 weeks preceding week 24, as recorded by participants in response to the global assessment question in the long-term diary. Participants were asked: “Please choose the response below that best describes the overall severity of your COVID-19 symptoms over the past 4 weeks,” with response options of “no symptoms,” “mild,” “moderate,” and “severe.” Participants selecting any response other than “no symptoms” were classified as having LC. This single global assessment question reflects participants' overall judgment of whether they were still experiencing COVID-19–related illness, and it is distinct from the assessment of 27 individual symptoms also recorded in the diary. This definition has been used consistently in prior publications from our group using the ACTIV-2/A5401 long-term diary and is supported by analyses supporting the internal validity of this definition [18, 24, 25].

### Potential Predictors of LC

Demographic and pre–COVID-19 clinical characteristics examined as potential predictors of risk of LC included age, sex, patient-reported racial identity (Black, White, or other), ethnicity (Hispanic/Latino or non-Hispanic/Latino), country of enrollment (non-US or US), body mass index (BMI) category (<25, 25 to <30, 30 to <35, or ≥35 [calculated as weight in kilograms divided by height in meters squared]), cigarette smoking status at study enrollment (current, former, or never), history of COVID vaccine prior to study entry, and presence of ≥1 comorbid condition conferring increased risk for severe COVID-19 (yes or no). In addition, clinical and biomarker characteristics examined during the acute COVID period (days 0–28) included total symptom score on day 0, presence of ≥1 symptom in the acute diary between days 22 and 28, serostatus on day 0, anterior nasal SARS-CoV-2 viral RNA (vRNA) level on day 0, AUC viral burden, and serum inflammatory and coagulation biomarkers on days 0 and 28. Receipt of first COVID-19 vaccination between study entry and week 24 was also evaluated in supplementary analyses.

### Statistical Analysis

Associations with log risk of LC were assessed using univariable and multivariable modified Poisson regression models with robust variance estimation [26]. Demographic and pre–COVID-19 clinical characteristics were first evaluated as predictors of LC in a multivariable model (base model). Building on the base model, we then evaluated whether symptom measures or biomarkers evaluated during the acute COVID-19 period were associated with risk of LC. Day 0 vRNA, D-dimer, and CRP had assay LLoQs and thus were modeled using 2 covariates: an indicator of <LLoQ versus ≥LLoQ, and quantitative value when ≥LLoQ. D-dimer, CRP, and ferritin were log-transformed to improve model fit. After the significant symptom measure and biomarker main effects were identified in multivariable analyses, effect modification by sex was evaluated via interaction terms with these covariates. Results are presented as risk ratios (RRs), 95% confidence intervals (CIs), and *P* values from Wald tests, with global *P* values provided for predictors with >2 categories. Statistical significance was based on a 2-sided 5% type I error rate without adjustment for multiple comparisons for this exploratory analysis. Analyses were performed using SAS software, version 9.4 (SAS Institute).

### Patient Consent Statement

The protocol was approved by a central institutional review board, Advarra (Pro00045266), with local institutional review board review and approval as required by sites, and by local ethics committees for non-US sites. All participants provided written informed consent.

### Role of the Funding Source

The study sponsor, the National Institutes of Health/National Institute of Allergy and Infectious Diseases Division of AIDS, participated in the design of the ACTIV-2/A5401 study and reviewed and approved the ACTIV-2/A5401 protocol prior to study initiation. Oversight and responsibility for data collection was delegated by the sponsor to PPD clinical research, a contract research organization.

## RESULTS

### Participant Characteristics

The analysis population included 545 participants who completed the long-term diary at week 24, comprising 78% of the 699 placebo recipients enrolled at sites without data integrity concerns between January and August 2021. Their median age was 44 years, 53% were female, 13% identified as Black/African American, and 53% as Hispanic/Latino (Table 1). The majority (83%) were enrolled in the United States, with the remainder enrolled in Argentina, South Africa, Mexico, and the Philippines. At enrollment, the median BMI was 29.3, 113 participants (21%) were current smokers, 317 (58%) reported ≥1 high-risk comorbid condition, and 83 (15%) had previously received ≥1 dose of a SARS-CoV-2 vaccine. At day 0, the median total symptom score was 11, the median vRNA was 3.7 log_10_ copies/mL, and 266 (52%) had positive anti-N and/or anti-S serostatus (Table 1).

**Table 1.** Characteristics of Participants Overall and by Long COVID Status at Week 24

Characteristic	Participants, No. (%)^a^		
	LC Absent (n = 471)	LC Present (n = 74)	Total (n = 545)
Demographic and pre–COVID-19 clinical characteristics			
Age, median (IQR), y	43 (34–54)	47 (38–56)	44 (35–54)
Sex			
Female	232 (49)	55 (74)	287 (53)
Male	239 (51)	19 (26)	258 (47)
Race			
White	381 (81)	56 (76)	437 (80)
Black or African American	61 (13)	9 (12)	70 (13)
Asian	17 (4)	1 (1)	18 (3)
American Indian or Alaska Native	0 (0)	1 (1)	1 (0)
Multiple	3 (1)	3 (4)	6 (1)
Other	9 (2)	4 (5)	13 (2)
Hispanic/Latino ethnicity	255 (54)	33 (45)	288 (53)
Country of enrollment			
Non-US	84 (18)	6 (8)	90 (17)
US	387 (82)	68 (92)	455 (83)
BMI, median (IQR)^b^	29.2 (25.5–33.9)	30.6 (25.6–35.0)	29.3 (25.5–34.1)
Cigarette smoking status			
Current	96 (20)	17 (23)	113 (21)
Former	55 (12)	9 (12)	64 (12)
Never	320 (68)	47 (64)	367 (67)
History of COVID vaccine before entry^c^	71 (15)	12 (16)	83 (15)
Reporting ≥1 high-risk comorbid condition^d^	269 (57)	48 (65)	317 (58)
Acute COVID clinical features			
Total symptom score at d 0, median (IQR)	10 (7–16)	12 (9–18)	11 (7–16)
Symptom presence from d 22 to d 28^e^			
Yes	211 (46)	48 (66)	259 (48)
No	251 (54)	25 (34)	276 (52)
COVID-19 AN RNA at d 0, median (IQR), log_10_ copies/mL^f^	3.6 (<LLoQ to 5.4)	4.3 (<LLoQ to 6.0)	3.7 (<LLoQ to 5.5)
Viral burden AUC, median (IQR)^g^	3.9 (0.0–15.3)	8.1 (0.5–25.7)	4.3 (0.0–16.1)
Acute COVID biomarkers			
Anti-N/anti-S serostatus at d 0^h^			
Positive	231 (52)	35 (51)	266 (52)
Negative	216 (48)	33 (49)	249 (48)
Fibrinogen, median (IQR), mg/dL			
d 0	368 (306–410) (n = 441)	363 (314–419) (n = 71)	366 (309–414.5) (n = 512)
d 28	306 (264.5–355) (n = 428)	335 (285–387.5) (n = 68)	313 (266–359) (n = 496)
aPTT, median (IQR), s			
d 0	30.9 (28.8–33.8) (n = 423)	29.7 (27.7–33.7) (n = 69)	30.8 (28.7–33.8) (n = 492)
d 28	31.3 (28.8–35.0) (n = 416)	30.8 (28.5–32.8) (n = 66)	31.2 (28.7–34.6) (n = 482)
PT-INR, median (IQR)			
d 0	1.0 (1.0–1.1) (n = 422)	1.0 (0.9–1.1) (n = 69)	1.0 (0.9–1.1) (n = 491)
d 28	1.0 (0.9–1.1) (n = 419)	1.0 (0.9–1.0) (n = 66)	1.0 (0.9–1.1) (n = 485)
D-dimer, median (IQR), ng/mL DDU			
d 0	<LLoQ (<LLoQ to 183) (n = 422)	141 (<LLoQ to 194) (n = 71)	<LLoQ (<LLoQ to 185) (n = 493)
d 28	<LLoQ (<LLoQ to 183) (n = 419)	122 (<LLoQ to 193) (n = 67)	<LLoQ (<LLoQ to 183) (n = 486)
CRP, median (IQR), mg/L			
d 0	5.4 (1.9–14.7) (n = 456)	5.7 (2.3–16.2) (n = 72)	5.4 (1.9–15.3) (n = 528)
d 28	2.2 (1.0–5.2) (n = 445)	3.7 (1.2–9.2) (n = 68)	2.4 (1.1–5.7) (n = 513)
LDH, median (IQR), U/L			
d 0	185 (160–218) (n = 450)	206.5 (160–261.5) (n = 72)	187 (160–225) (n = 522)
d 28	177 (155–202) (n = 442)	177 (159–204.5) (n = 68)	177 (155–203) (n = 510)
Ferritin, median (IQR), μg/L			
d 0	176 (75–377) (n = 449)	198.5 (55–355) (n = 70)	181 (72–373) (n = 519)
d 28	111 (46–236) (n = 444)	122.5 (33.5–232.5) (n = 68)	114 (45–236) (n = 512)

Abbreviations: AN, anterior nasal; anti-N, antinucleocapsid; anti-S, antispike; aPTT, activated partial thromboplastin time; AUC, area under the curve; BMI, body mass index; COVID-19, coronavirus disease 2019; CRP, C-reactive protein; d, day; DDU, D-dimer units; IQR, interquartile range; LC, long COVID; LDH, lactate dehydrogenase; LLoQ, lower limit of quantification; PT-INR, prothrombin time/international normalized ratio.

^a^Data represent no. (%) of participants unless otherwise specified. Data were not collected for all participants. BMI was not collected for 2 participants, the total symptom score at day 0 was missing for 36, and the viral burden AUC was missing for 161. Sample sizes are provided in the table for each biomarker.

^b^BMI calculated as weight in kilograms divided by height in meters squared.

^c^Any single dose of COVID-19 vaccine regardless of brand.

^d^High-risk comorbid conditions include active cancer, moderate to severe asthma, chronic kidney disease, chronic liver disease, history of cirrhosis, chronic lung disease, current smoker, cardiovascular disease, diabetes mellitus, hypertension, treatment with biologics/immunomodulators/cancer chemotherapy within 90 days of entry, human immunodeficiency virus with CD4 cell count <200/μL, receipt of corticosteroids within 30 days of entry, and obesity. Smoking and BMI were included both as individual variables and within the composite definition of “high-risk comorbid condition.”

^e^Symptom presence defined as “yes” if ≥1 symptom reported in daily diary between day 22 and day 28 and as “no” if no symptoms reported on any day during this period.

^f^AN swab samples were not collected for 120 participants after sample collection was discontinued from the study in a later protocol version. Day 0 AN swabs were unavailable for another 41 participants due to thawed specimens or other reasons. The assay LLoQ is 2.00 log_10_ copies/mL.

^g^The viral burden AUC is the AUC of log_10_ severe acute respiratory syndrome coronavirus 2 (SARS-CoV-2) RNA values over time above the assay LLoQ (2 log_10_ copies/mL) between day 0 and day 28.

^h^Serostatus defined as positive if anti-N and/or anti-S positive and as negative if both negative.

### LC Outcomes

At week 24, 74 participants (14%) met the study LC definition (Table 1). Each of the 27 individual symptoms assessed in the long-term diary was reported with greater frequency among participants reporting LC at week 24 than among those who did not (Figure 1). This was also seen when symptoms were categorized by affected body system and evaluated separately by sex (Supplementary Figure 1). Female participants who reported LC reported more symptoms of any severity across all body systems except upper respiratory, where male participants reporting LC reported a higher frequency (58% vs 44%). Female participants reporting LC also generally reported more symptoms of moderate or severe severity than male participants reporting LC, particularly for dermatologic (29% vs 11%, respectively) and neurocognitive symptoms (25% vs 16%).

**Figure 1.**
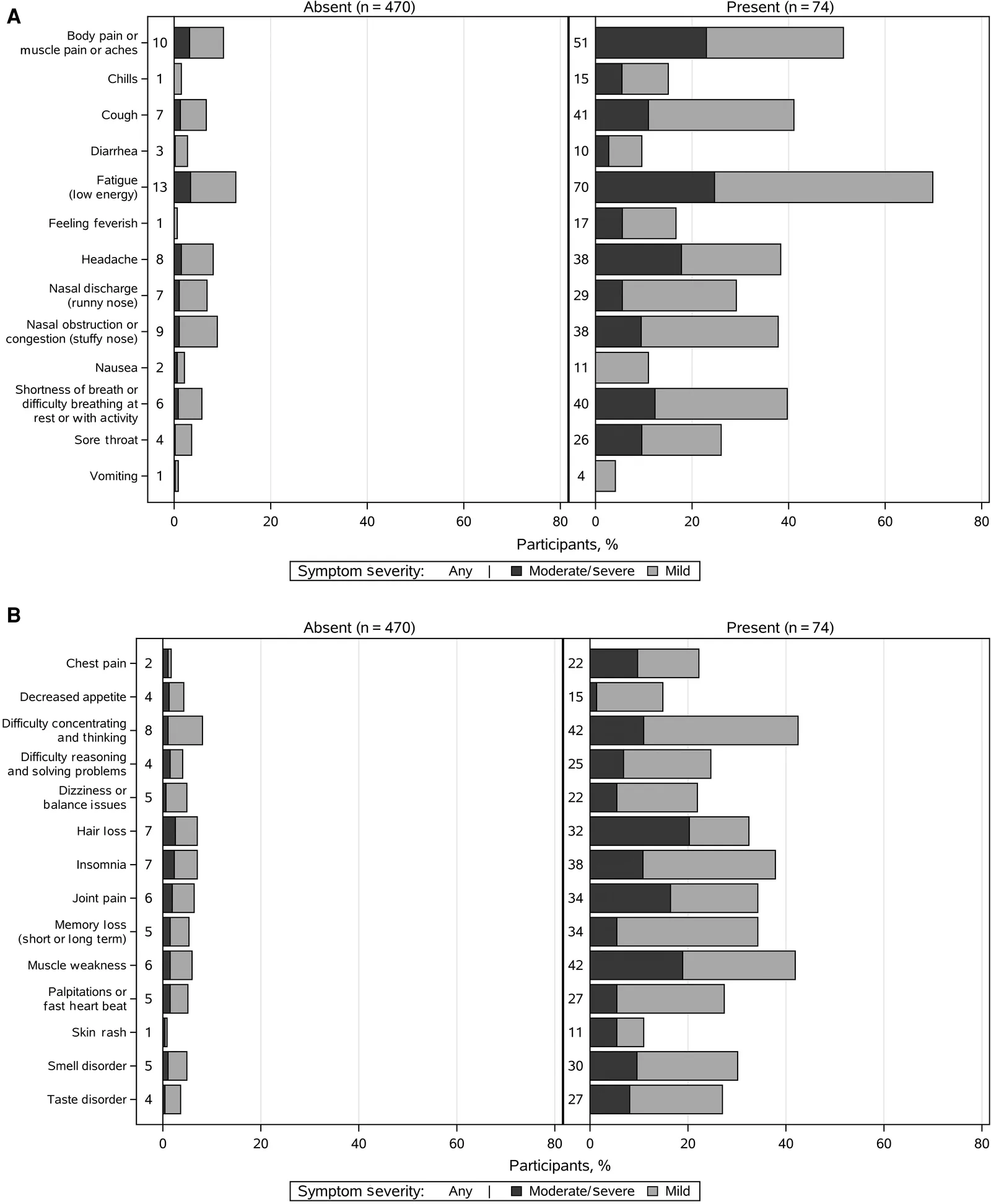
Frequency and severity of 13 symptoms that were also assessed in the acute symptom diary (*A*) and 14 additional long COVID (LC)–related symptoms (*B*) among participants who reported the presence (*right-hand panels*) or absence (*left-hand panels*) of LC (based on self-report of overall coronavirus disease 2019 symptoms on global assessment). The global assessment question and the individual symptom items were both part of the long-term diary completed at week 24. However, LC classification was based solely on the global assessment question and not derived from the individual symptom items. One female participant without reported LC at week 24 was missing all entries on individual symptoms.

### Demographic and Pre–COVID-19 Clinical Characteristics

Only female sex (adjusted RR [aRR], 2.64 [95% CI, 1.58–4.42]; *P* < .001) and non-US country of enrollment (0.29 [.12–.66]; *P* = .003) were significant predictors of LC in both univariable and multivariable models (Table 2). In multivariable but not univariable analysis, race was a statistically significant predictor of LC (*P* = .03); however, this finding reflected higher risk in the “other” race category, a small and heterogeneous group.

**Table 2.** Risk Ratios for Long COVID for Demographic and Pre–Coronavirus Disease 2019 Clinical Characteristics (N=545)

Demographic or Pre–COVID-19 Characteristic	Category	Unadjusted		Adjusted	
		RR (95% CI)	*P* Value	aRR^a^ (95% CI)	*P* Value
Age (per 10 y higher)		1.13 (.97–1.31)	.11	1.12 (.95–1.33)	.19
Sex	Female	2.60 (1.59–4.26)	<.001	2.64 (1.58–4.42)	<.001
	Male	Reference		Reference	
Country of enrollment	Non-US	0.45 (.20–1.00)	.049	0.29 (.12–.66)	.003
	US	Reference		Reference	
Race	Black or African American	1.00 (.52–1.94)	.15	1.03 (.55–1.92)	.03
	Other	1.85 (.99–3.44)		2.40 (1.23–4.68)	
	White	Reference		Reference	
Ethnicity	Hispanic or Latino	0.72 (.47–1.10)	.13	0.78 (.47–1.29)	.34
	Not Hispanic or Latino	Reference		Reference	
BMI category^b^	≥35	1.21 (.65–2.27)	.66	1.10 (.56–2.15)	.73
	30 to <35	1.13 (.60–2.11)		1.07 (.57–2.02)	
	25 to <30	0.86 (.46–1.59)		0.81 (.42–1.55)	
	<25	Reference		Reference	
Cigarette smoking status	Current	1.17 (.70–1.96)	.82	1.17 (.65–2.11)	.82
	Former	1.10 (.57–2.13)		0.94 (.48–1.86)	
	Never	Reference		Reference	
History of COVID-19 vaccine before entry	Yes	1.08 (.61–1.91)	.80	1.21 (.62–2.36)	.57
	No	Reference		Reference	
Reporting ≥1 high-risk comorbid condition	Yes	1.33 (.85–2.07)	.21	1.49 (.80–2.77)	.21
	No	Reference		Reference	

Abbreviations: aRR, adjusted risk ratio; CI, confidence interval; COVID-19, coronavirus disease 2019; RR, risk ratio.

^a^aRRs were obtained from a single multivariable model that included all the variables shown, using data from the 542 participants with complete data for all variables.

^b^BMI calculated as weight in kilograms divided by height in meters squared.

### Total Symptom Score at Day 0

Higher total symptom score at day 0 was significantly associated with higher risk of LC in univariable analysis and remained a significant predictor when added to the multivariable base model, which included the demographic and pre–COVID-19 clinical characteristics (aRR, 1.37 per 10-point higher score [95% CI, 1.03–1.81]) (Table 3). The increased risk seen with female sex remained in this multivariable model after adjustment for total symptom score (aRR, 2.37 [95% CI, 1.43–3.96]; *P* < .001), suggesting that the extent of symptoms at day 0 did not explain the risk difference by sex.

**Table 3.** Risk Ratios for Long COVID for Clinical Characteristics and Biomarkers Evaluated During Acute Coronavirus Disease 2019 Follow-up (Study Days 0–28)

Acute COVID-19 Characteristic	Unadjusted		Adjusted	
	RR (95% CI)	*P* Value	aRR^a^ (95% CI)	*P* Value
Total symptom score at d 0 (per 10 points higher)	1.53 (1.14–2.03)	.004	1.37 (1.03–1.81)	.03
Serostatus at d 0^b^				
Positive	0.99 (.64–1.55)	.99	0.98 (.60–1.59)	.93
Negative	Reference		Reference	
Anterior nasal vRNA at d 0 (per 1 log_10_ copies/mL higher)^c^	1.13 (.97–1.32)	.27	1.10 (.94–1.29)	.24
Viral burden AUC (per 5 log_10_ copies/mL ⋅ d higher)^d^	1.06 (1.00–1.12)	.04	1.07 (1.01–1.13)	.02
Symptom presence from d 22 to d 28^e^				
Yes	2.05 (1.30–3.22)	.002	1.69 (1.08–2.66)	.02
No	Reference		Reference	

Abbreviations: aRR, adjusted risk ratio; AUC, area under the curve; CI, confidence interval; COVID-19, coronavirus disease 2019; RR, risk ratio; vRNA, viral RNA.

^a^aRRs were obtained from a single multivariable model that included the variable shown as well as the demographic and pre–COVID-19 variables shown in Table 2.

^b^Serostatus defined as positive if antinucleocapsid and/or antispike serostatus positive; negative if both negative.

^c^Model with quantitative log_10_ anterior nasal severe acute respiratory syndrome coronavirus 2 (SARS-CoV-2) RNA as covariate also included an indicator variable for values less than the assay lower limit of quantification (LLoQ) (model included 384 participants with available anterior nasal samples on day 0; the study did not require collection of these samples among participants enrolled later in the study).

^d^Viral burden AUC is the AUC of log_10_ SARS-CoV-2 RNA values over time above the assay LLoQ (2 log_10_ copies/mL) between days 0 and 28.

^e^Symptom presence was defined as “yes” if ≥1 symptom was reported in the daily diary between days 22 and 28, and “no” if no symptoms were reported on any day during this period.

Although the distribution of total symptom scores at day 0 was similar for female and male participants (median [interquartile range (IQR)], 11 [8–16] and 10 [7–15], respectively), we found some evidence that the association between risk of LC and total symptom score varied by sex. There was a trend of increasing risk of LC with higher total symptom scores among female participants (RR, 1.68 per 10-point higher score [95% CI, 1.23–2.30]) but any association was inconclusive in male participants (0.85 per 10-point higher score [.47–1.55]; *P* = .048 for interaction between sex and total symptom score) (Figure 2), suggesting that the association between LC and total symptom score may vary by sex. In multivariable analysis, adjusting for demographic and pre–COVID-19 clinical characteristics, the association remained, but the interaction was not statistically significant (aRR, 1.55 per 10-point higher score [95% CI, 1.14–2.12] for female and 0.84 [.43–1.63] for male participants; interaction *P* = .11).

**Figure 2.**
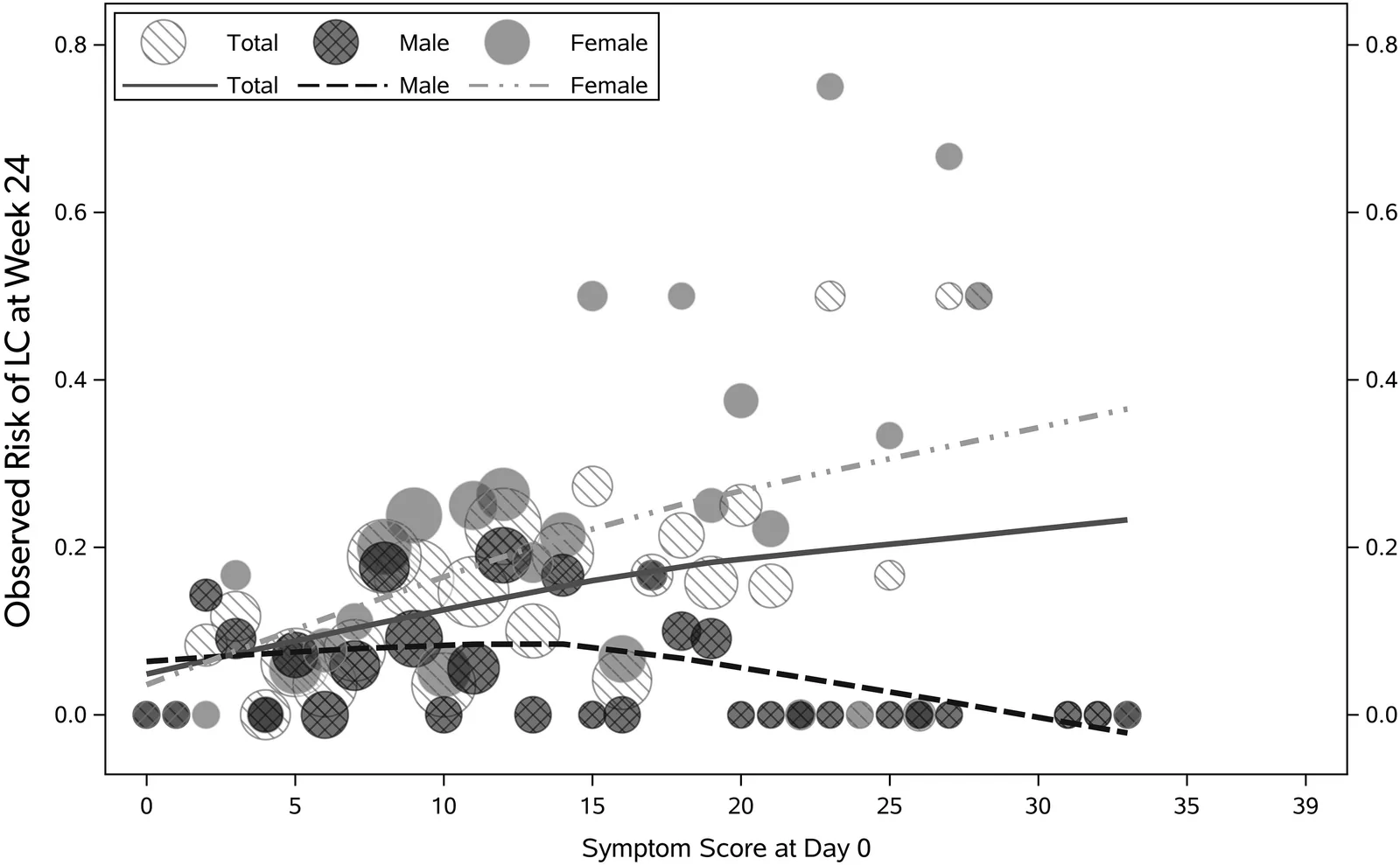
Observed risk of long COVID (LC) at week 24 by total symptom score at study day 0, by sex and in the total study population. The observed risk is calculated as the proportion of participants with LC at week 24 for each possible value of total symptom score. Bubble sizes are proportional to the number of participants with each given value of the total symptom score. The Loess method was used to fit a smooth curve to the data, with weighting by the number of participants for each value of total symptom score.

### Anti–SARS-CoV-2 Serostatus and Nasal vRNA

There was no evidence that anti–SARS-CoV-2 serostatus on day 0 was a predictor of LC in univariable or multivariable models (aRR for seropositive vs seronegative, 0.98 [95% CI, .60–1.59]; *P* = .93) (Table 3). Nasal vRNA on day 0 was not associated with risk of LC (aRR per 1 log_10_ copies/mL higher vRNA, 1.10 [95% CI, .94–1.29]; *P* = .24); however, higher viral burden between days 0 and 28 (vRNA AUC above the assay LLoQ) was a significant predictor in univariable analysis. Participants with LC had approximately twice the median viral burden of those without (median vRNA AUC, 8.1 vs 3.9 log_10_ copies/mL ⋅ d, respectively). In multivariable analysis adjusted for demographic and pre–COVID-19 clinical characteristics, this association persisted (aRR, 1.07 per 5 log_10_ copies/mL ⋅ d higher [95% CI, 1.01–1.13]; *P* = .02). To evaluate whether viral burden might explain the observed sex difference in LC risk, the effect of female sex on LC was examined after adjustment for vRNA AUC. This effect of female sex persisted (aRR, 2.68 [95% CI, 1.48–4.86]; *P* = .001). There was no evidence that the association of risk of LC with vRNA AUC varied by sex (interaction *P* = .92).

### Late Acute Symptoms (Days 22–28)

Among 259 participants reporting symptoms on ≥1 day during the last week of acute COVID follow-up (study days 22–28), 48 (19%) had LC at week 24. This compares with 25 (9%) of the 276 who did not have late acute symptoms (RR, 2.05 [95% CI, 1.30–3.22]; *P* = .002) (Table 3). This effect was attenuated in multivariable analysis adjusted for demographic and pre–COVID-19 clinical characteristics (aRR, 1.69 [95% CI, 1.08–2.66]; *P* = .02) (Table 3). To evaluate whether late acute symptoms might explain the observed sex difference in LC risk, the effect of female sex on LC was examined after adjustment for the presence of late acute symptoms. This effect of female sex persisted (aRR, 2.45 [95% CI, 1.46–4.12]; *P* < .001). There was no evidence that the association of risk of LC with presence of late acute symptoms varied by sex (interaction *P* = .73).

### Inflammatory and Coagulation Biomarkers During Acute COVID

There was no significant association between fibrinogen, aPTT, PT-INR, D-dimer, CRP, LDH, or ferritin levels at day 0 and risk of LC in univariable or multivariable analysis adjusted for demographic and pre–COVID-19 clinical characteristics (*P* > .05) (Figure 3 and Supplementary Table 1). In both univariable and multivariable analyses, aPTT, D-dimer, LDH and ferritin levels at day 28 were also not significantly associated with risk of LC (*P* > .05). PT-INR at day 28 was significant in univariable analysis, though the association was attenuated and not significant in multivariable analysis. There was an increased risk of LC with higher CRP at day 28 in univariable and multivariable analysis (aRR, 1.12 per 0.2 log_10_ mg/L higher [95% CI, 1.00–1.25]; *P* = .04) (Figure 3). This association did not differ significantly by sex (interaction *P* = .14). CRP levels were lower at day 28 (median, 2.39 mg/L [IQR, 1.10–5.70]) than at day 0 (5.40 mg/L [1.90–15.30]).

**Figure 3.**
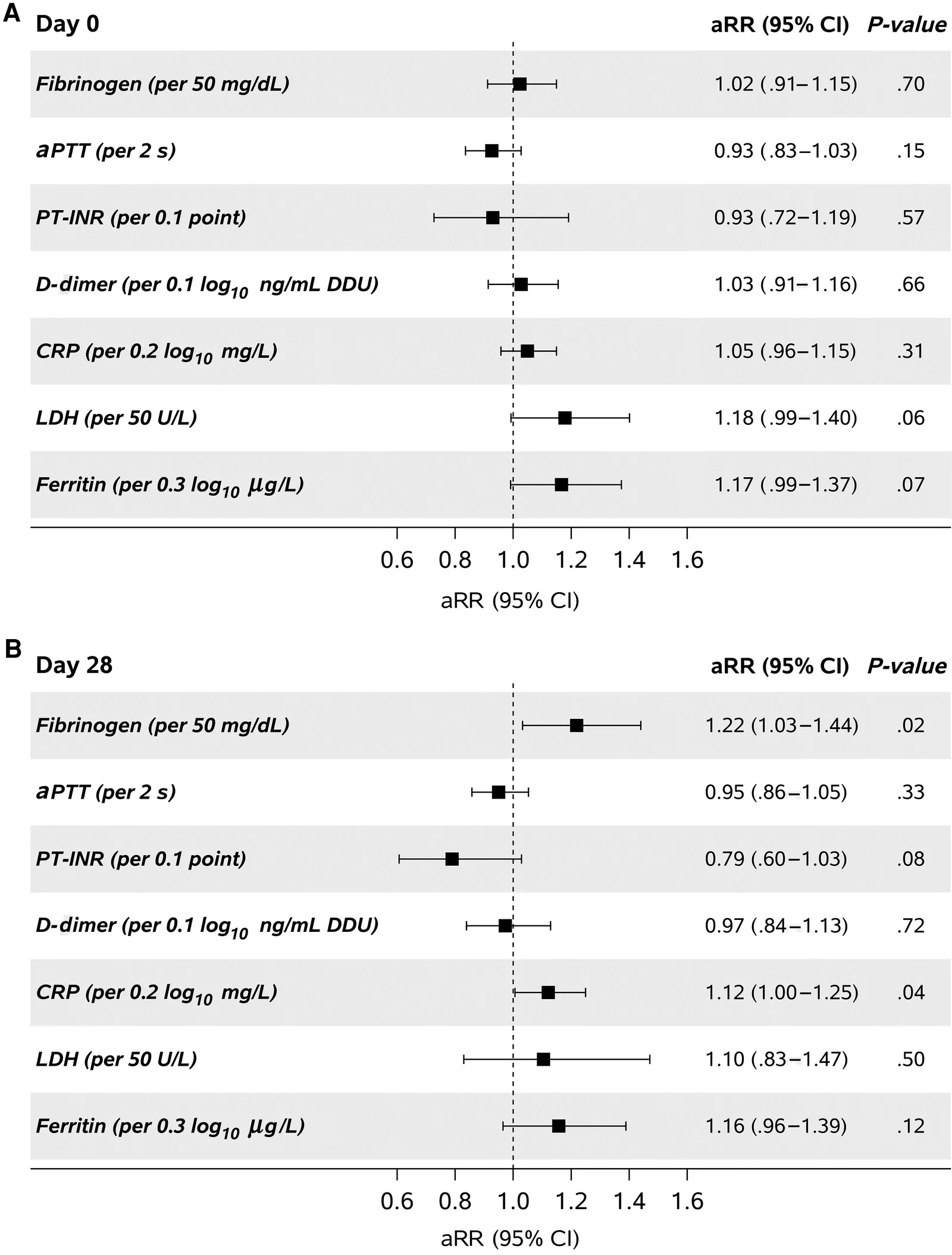
Adjusted risk ratios (aRRs) for long COVID for inflammatory and coagulation biomarkers measured at days 0 (*A*) and 28 (*B*). aRRs were obtained from multivariable models adjusted for demographic and pre–coronavirus disease 2019 clinical characteristics shown in Table 2. Abbreviations: aPTT, activated partial thromboplastin time; CI, confidence interval; CRP, C-reactive protein; DDU, D-dimer units; LDH, lactate dehydrogenase; PT-INR, prothrombin time/international normalized ratio.

Higher fibrinogen levels at day 28 were also associated with an increased risk of LC in univariable and multivariable analysis (aRR, 1.22 per 50 mg/dL higher [95% CI, 1.03–1.44]; *P* = .02) (Figure 3). The association of risk of LC with fibrinogen level at day 28 differed by sex in unadjusted analysis (interaction *P* = .02) (Figure 4). This effect modification persisted in multivariable analysis, reflecting evidence that higher fibrinogen levels at day 28 were associated with higher risk of LC among male participants (aRR, 1.56 per 50 mg/dL higher [95% CI, 1.23–2.00]), whereas there was no clear association among female participants (1.05 [.87–1.27]; interaction *P* = .01). This effect modification was not observed for fibrinogen levels at day 0 (Figure 4). Male and female participants had similar fibrinogen levels at day 0 (median [IQR], 365 [313–440] mg/dL for male vs 368 [303–405] mg/dL for female participants; *P* = .15), but male participants had greater decreases by day 28 overall, resulting in significantly lower values at day 28 than in female participants (286 [250–341] mg/dL for male vs 333 [286–368] mg/dL for female participants; *P* < .001).

**Figure 4.**
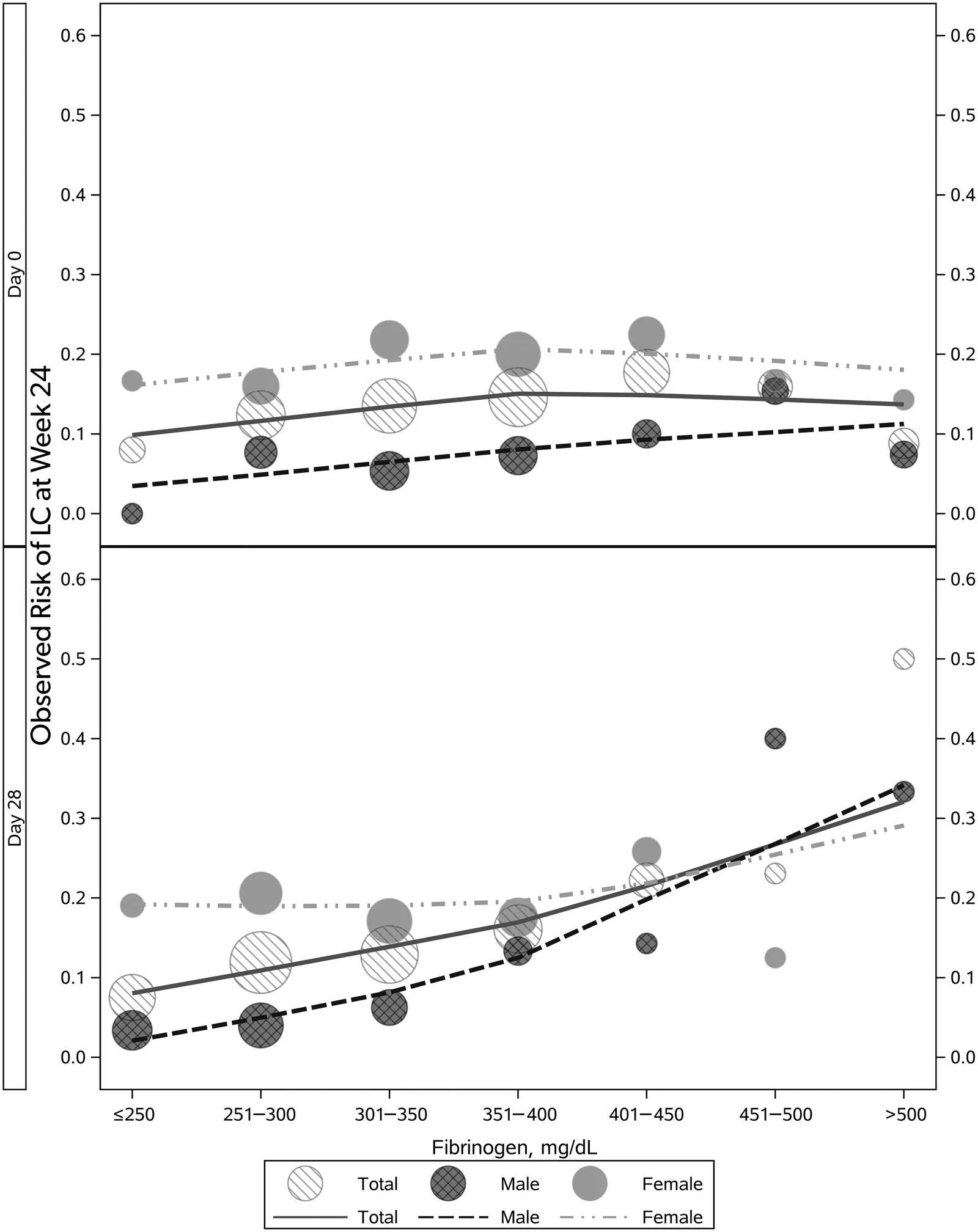
Observed risk of long COVID (LC) for categories of fibrinogen levels on days 0 and 28, by sex. Bubble sizes are proportional to number of participants with each level of fibrinogen.

The difference in risk of LC between female and male participants persisted even with adjustment for the 7 inflammatory and coagulation biomarkers evaluated at days 0 and 28: the aRR for sex from multivariable models was ≥2.40 in all analyses, compared with the unadjusted RR of 2.60.

### Postentry COVID Vaccination

Eighty-one participants (15%) received their first COVID vaccine between entry and week 24, most within 16 weeks of study entry; 70% of participants had not received a SARS-CoV-2 vaccine by week 24. There was no significant association between COVID vaccination and LC (Supplementary Table 2).

## DISCUSSION

We identified several key predictors of increased risk of LC in this prospective evaluation of participants who received placebo in the ACTIV-2 trial of nonhospitalized adults with mild-to-moderate COVID-19. These included female sex, US enrollment, higher total symptom score at day 0 (early during acute COVID-19), viral burden as measured by the AUC above the LLoQ of the assay over the acute disease period, and elevated CRP and fibrinogen levels at day 28. By focusing on the placebo group, our findings provide important insights into the natural history and potential mechanisms of LC development. This analysis is strengthened by the concurrent real-time measurement of virologic and inflammatory biomarkers within a randomized trial with prespecified LC end points.

The strong association of female sex with LC risk, which persisted even with adjustment for demographic and pre–COVID-19 clinical characteristics such as BMI category, smoking status and comorbid conditions, aligns with previous reports [11, 16, 19, 27]. We further found that it persisted after adjustment for serostatus and viral burden as well as for several biomarkers of inflammation and coagulation.

Disturbances of inflammatory and coagulation pathways have been implicated in the pathogenesis of SARS-CoV-2 infection and LC [28–30]. Underlying mechanisms proposed include endothelial damage [31], the presence of prothrombotic autoantibodies [32, 33], and circulation of aggregates of platelets and clotting proteins (microclots) [34]. The association we observed between elevated CRP and fibrinogen at day 28 and LC risk is consistent with a role for persistent inflammation, while the association with fibrinogen at day 28 may also implicate coagulation pathways, though this rests on a single marker at a single time point. The observation that CRP and fibrinogen levels at day 28, but not day 0, were associated with LC risk may suggest that the trajectory of inflammatory resolution during acute illness is relevant to subsequent LC risk. However, this interpretation is speculative and requires validation.

Other biological mechanisms may therefore underlie sex-based differences in SARS-CoV-2 infection trajectories and LC risk. These include sex-specific variations in innate and adaptive immunity [35, 36], differential patterns of autoantibody production [37], and distinct hormonal influences on immune function [38, 39]. However, adjustment for inflammatory and coagulation biomarkers in our analysis did not substantially attenuate the association between female sex and LC risk, suggesting that these measured pathways do not fully explain the observed sex differences.

We found that higher viral burden in nasal samples between days 0 and 28, assessed by the vRNA AUC (above the assay LLoQ), was a significant predictor of higher risk of LC. This provides support to other studies finding that higher and more persistent viral levels during acute infection might be associated with greater risk of LC [40, 41], possibly reflecting infections that persist longer in tissues [42, 43] or more intense immune responses [44, 45].

The increased risk we observed in LC among US participants warrants careful interpretation. This association might reflect differences in healthcare access, symptom reporting patterns, circulating SARS-CoV-2 variants, or environmental factors, rather than biological differences in disease risk. Future studies should examine whether similar geographic variations exist in other cohorts.

We did not find statistically significant associations between LC risk and factors previously reported as predictors in other prospective cohorts, including advanced age, active smoking or the presence of comorbid conditions [11, 16, 46]. However, the direction of trends in associations with these factors was consistent with other studies.

With the caveat that we evaluated multiple predictor variables for possible effect modification by sex, we identified possible sex-specific differences in associations between LC risk and symptom burden at day 0 as well as fibrinogen level at day 28. The association between risk of LC and total symptom score at day 0 differed between women and men, with women showing a stronger association between initial symptom burden and risk of LC. This observation suggests that early symptom burden may be a more robust predictor of LC risk in women than in men. Conversely, the association between risk of LC and fibrinogen at day 28 differed between women and men, with men showing a stronger association between higher fibrinogen levels and higher risk of LC, though the mechanisms underlying this remain unclear.

Our study has several limitations. The introduction of the long-term diary partway through the trial means that our analysis population represents a subset, though a large majority, of all placebo recipients. Of those eligible, 78% completed the week 24 diary and were included in the analysis. The direction of potential bias from this is uncertain. If participants who had recovered fully were less motivated to complete follow-up diaries, the observed LC prevalence of 14% may be an overestimate. Conversely, if those with more burdensome ongoing symptoms were less able to complete assessments, it may be an underestimate. The exploration of sex-specific differences related to the risk of LC is limited by the small number of male participants who experienced LC in our analysis population. The predominance of participants identifying as White, though the cohort also included substantial Hispanic/Latino representation, may also limit the generalizability of our findings to other racial groups.

As enrollment was restricted to nonhospitalized adults with symptomatic mild-to-moderate COVID-19, our findings may not generalize to individuals with asymptomatic infection or those with severe disease requiring hospitalization, both of whom have been reported to develop LC. Although enrollment occurred primarily before widespread vaccination, vaccination status including receipt of COVID-19 vaccine after study entry was captured and evaluated in relation to LC risk. Neither pre-enrollment nor postenrollment vaccination was significantly associated with LC risk. The prevaccination timing of the study may better reflect natural LC pathogenesis without vaccine-induced immunologic modifications. While our biomarker analyses suggest potential mechanisms, we cannot establish causality. Finally, our LC definition, based on participants' global self-assessment of overall COVID-19–related symptom severity at week 24, differs from established international case definitions [4]. These differences should be considered when comparing LC prevalence across studies.

These findings have important implications for both clinical care and research. The substantially higher risk of LC among women found in our study (and other studies), which persisted after adjustment for other demographic and clinical variables as well as multiple biomarkers, highlights the need for further research to advance understanding of this difference. The association with viral burden during the acute disease period and with day 28 inflammatory markers suggests a potential window for therapeutic intervention to prevent LC. In addition, our results support investigation of anticoagulation or anti-inflammatory strategies for LC prevention or treatment. In conclusion, our findings suggest that the pathophysiology of LC may differ between men and women, with implications for both future mechanistic studies and therapeutic approaches.

## Supplementary Material

ofag577_Supplementary_Data

## Supplementary Data

Supplementary materials are available at *Open Forum Infectious Diseases* online. Consisting of data provided by the authors to benefit the reader, the posted materials are not copyedited and are the sole responsibility of the authors, so questions or comments should be addressed to the corresponding author.
