# Supplementary material for: Predictors of Long COVID in Adults With Untreated Mild-to-Moderate Coronavirus Disease 2019
Source: Open Forum Infect Dis. 2026 Nov 13;13(11):ofag577. doi: 10.1093/ofid/ofag577 (PMC13759973; doi:10.1093/ofid/ofag577)
Supplement: Supplementary file 1 [file ofag577_supplementary_data.docx]

**Supplemental Material**

**Supplemental Methods: Serology, Inflammatory and Coagulation Biomarker Assays**

Supplemental Table 1: Risk Ratios of Long COVID for each Inflammatory and Coagulation Biomarker Measured at Day 0 and Day 28

Supplemental Table 2: Association between COVID-19 Vaccination Status and Risk of Long COVID at Week 24

Supplemental Figure 1: Severity of 27 Long-term Symptoms (Categorized by Symptom Category) on Long-Term Diary at Week 24 by Long COVID Status at Week 24 by Sex: (A) Total, (B) Females, and (C) Males by Sex at Birth

**Supplemental Methods: Serology, Inflammatory and Coagulation Biomarker Assays**

Anti-nucleocapsid (N) and anti-Spike (S) binding antibodies (Elecsys Anti-SARS-CoV-2 and Elecsys Anti-SARS-CoV-2 S, Roche Diagnostics) in serum were measured on day 0 by a central laboratory (University of Washington). Serum inflammatory and coagulation markers including C-reactive protein (CRP) (Tina-quant® C-Reactive Protein IV, cobas® analyzer, Roche Diagnostics), ferritin (Elecsys® Ferritin, cobas® analyzer, Roche Diagnostics), D-dimer (HemosIL® D-dimer HS, ACL TOP 500 analyzer, Instrumentation Laboratory), lactate dehydrogenase (LDH) (LDHI2, cobas® analyzer, Roche Diagnostics), prothrombin time (PT)/international normalized ratio (INR) (HemosIL® RecombiPlasTin 2G, ACL TOP 500 analyzer, Instrumentation Laboratory), activated partial thromboplastin time (aPTT) (HemosIL® SynthASil, ACL TOP 500 analyzer, Instrumentation Laboratory), and fibrinogen (HemosIL® Fibrinogen-C, ACL TOP 500 analyzer, Instrumentation Laboratory) were measured in real time by a central clinical laboratory (PPD® Laboratory Services Global Central Labs) at days 0 and 28, per the manufacturers’ protocols.

**Supplemental Table 1**: Risk Ratios of Long COVID for each Inflammatory and Coagulation Biomarker Measured at Day 0 and Day 28

|  | **Unadjusted** | | | | **Adjusted** | | | |
| --- | --- | --- | --- | --- | --- | --- | --- | --- |
| **Inflammatory/Coagulation Markers** | RR | 95% CI | P-value | Global P-Value | RR | 95% CI | P-value | Global P-Value |
| Fibrinogen (per 50 mg/dL higher) |  |  |  |  |  |  |  |  |
| Day 0 | 1.00 | (0.91, 1.11) | 0.93 | 0.93 | 1.02 | (0.91, 1.15) | 0.70 | 0.70 |
| Day 28 | 1.28 | (1.11, 1.46) | <0.001 | <0.001 | 1.22 | (1.03, 1.44) | 0.021 | 0.021 |
| Activated Partial Thromboplastin Time  (per 2 seconds higher) |  |  |  |  |  |  |  |  |
| Day 0 | 0.92 | (0.82, 1.02) | 0.11 | 0.11 | 0.93 | (0.83, 1.03) | 0.15 | 0.15 |
| Day 28 | 0.95 | (0.87, 1.04) | 0.29 | 0.29 | 0.95 | (0.86, 1.05) | 0.33 | 0.33 |
| Prothrombin Intl. Normalized Ratio  (per 0.1 point higher) |  |  |  |  |  |  |  |  |
| Day 0 | 0.83 | (0.63, 1.10) | 0.19 | 0.19 | 0.93 | (0.72, 1.19) | 0.57 | 0.57 |
| Day 28 | 0.71 | (0.54, 0.93) | 0.014 | 0.014 | 0.79 | (0.60, 1.03) | 0.082 | 0.082 |
| D-Dimer  (per 0.1 log10 ng/mL DDU higher)^1^ |  |  |  |  |  |  |  |  |
| Day 0 | 1.02 | (0.90, 1.15) | 0.77 | 0.44 | 1.03 | (0.91, 1.16) | 0.66 | 0.47 |
| Day 28 | 0.93 | (0.80, 1.07) | 0.28 | 0.26 | 0.97 | (0.84, 1.13) | 0.72 | 0.82 |
| C Reactive Protein  (per 0.2 log10 mg/L higher)^1^ |  |  |  |  |  |  |  |  |
| Day 0 | 1.04 | (0.96, 1.12) | 0.37 | 0.46 | 1.05 | (0.96, 1.15) | 0.31 | 0.52 |
| Day 28 | 1.15 | (1.03, 1.30) | 0.015 | 0.029 | 1.12 | (1.00, 1.25) | 0.042 | 0.074 |
| Lactate Dehydrogenase  (per 50 U/L higher) |  |  |  |  |  |  |  |  |
| Day 0 | 1.14 | (0.98, 1.32) | 0.091 | 0.091 | 1.18 | (0.99, 1.40) | 0.064 | 0.064 |
| Day 28 | 1.09 | (0.84, 1.42) | 0.51 | 0.51 | 1.10 | (0.83, 1.47) | 0.50 | 0.50 |
| Ferritin  (per 0.3 log10 mcg/L higher) |  |  |  |  |  |  |  |  |
| Day 0 | 1.01 | (0.90, 1.14) | 0.84 | 0.84 | 1.17 | (0.99, 1.37) | 0.067 | 0.067 |
| Day 28 | 0.99 | (0.87, 1.13) | 0.87 | 0.87 | 1.16 | (0.96, 1.39) | 0.12 | 0.12 |

RR: Risk Ratio; aRR: adjusted Risk Ratio (obtained from a single multivariable model which included the variable shown as well as the demographic and pre-COVID variables shown in Table 2); CI: Confidence Interval.

^1^ Models with D-Dimer or C Reactive Protein as covariate also included an indicator variable for values less than the assay lower limit of quantification

**Supplemental Table** **2**: Risk Ratios for LC for COVID-19 Vaccination Status

|  |  |  | **Unadjusted** | | |  | **Adjusted** | | |
| --- | --- | --- | --- | --- | --- | --- | --- | --- | --- |
|  |  | **Prop. with LC** | **RR** | **95% CI** | **P-value** | **Prop. with LC** | **aRR** | **95% CI** | **P-value** |
| Receipt of Any¹ COVID-19 Vaccine | After Entry, by Week 24 | 16/81 (20%) | 1.64 | (0.98, 2.74) | 0.17 | 16/80 (20%) | 1.69 | (1.01, 2.83) | 0.12 |
|  | Prior to Entry | 12/83 (14%) | 1.20 | (0.66, 2.16) |  | 12/83 (14%) | 1.42 | (0.73, 2.78) |  |
|  | None | 46/381 (12%) |  | ref |  | 45/379 (12%) |  | ref |  |

RR: Risk Ratio; aRR: adjusted Risk Ratio (obtained from a single multivariable model which included the variable shown as well as the demographic and pre-COVID variables shown in Table 2, except for history of COVID vaccination at study entry, and using data from the 542 participants with complete data for all variables); CI: Confidence Interval; Ref: Reference category for comparisons.

¹ Any refers to any single dose of COVID-19 vaccination regardless of brand. Participants with vaccination after Entry and by Week 24 did not receive any vaccination prior to Entry.

**Supplemental Figure 1.** Severity of 27 Long-term Symptoms (Categorized by Symptom Category) on Long-Term Diary at Week 24 by Long COVID Status at Week 24 by Sex: (A) Total, (B) Females, and (C) Males by Sex at Birth

(A) Total

(B) Females

(C) Males

One female participant without reported Long COVID at week 24 was missing all entries on individual symptoms.

Symptom categories:

- Cardiopulmonary/Cardiothoracic: 'cough', 'shortness of breath or difficulty breathing', 'palpitations or fast heartbeat', and 'chest pain'.
- Constitutional: 'feeling feverish', 'chills', 'fatigue (low energy) ', and 'decreased appetite'.
- Dermatologic: 'hair loss' and 'skin rash'.
- Gastrointestinal: 'diarrhea', 'nausea', and 'vomiting'.
- Musculoskeletal: 'body pain or muscle pain or aches', 'muscle weakness', and 'joint pain'.
- Neurocognitive: 'headache', 'insomnia', 'difficulty with concentration and thinking', 'difficulty with reasoning and solving problems', 'dizziness/balance issues' and 'memory loss (short or long term)'.
- Sensory: 'smell disorder', 'taste disorder'.
- Upper respiratory: 'sore throat', 'nasal obstruction or congestion (stuffy nose)', and 'nasal discharge (runny nose)'.
